# Biofilm production by the multidrug-resistant fungus *Candida haemulonii* is affected by aspartic peptidase inhibitor

**DOI:** 10.3934/microbiol.2025012

**Published:** 2025-03-21

**Authors:** Joice Cavalcanti Lima, Lívia de Souza Ramos, Pedro Fernandes Barbosa, Iuri Casemiro Barcellos, Marta Helena Branquinha, André Luis Souza dos Santos

**Affiliations:** 1 Laboratório de Estudos Avançados em Microrganismos Emergentes e Resistentes (LEAMER), Departamento de Microbiologia Geral, Instituto de Microbiologia Paulo de Góes (IMPG), Universidade Federal do Rio de Janeiro (UFRJ), Rio de Janeiro, RJ, Brazil; 2 Programa de Pós-Graduação em Ciências (Microbiologia), Instituto de Microbiologia Paulo de Góes (IMPG), Universidade Federal do Rio de Janeiro (UFRJ), Rio de Janeiro, RJ, Brazil; 3 Programa de Pós-Graduação em Bioquímica (PPGBq), Instituto de Química, Universidade Federal do Rio de Janeiro (UFRJ), Rio de Janeiro, RJ, Brazil; 4 Instituto Federal de Educação, Ciência e Tecnologia do Rio de Janeiro (IFRJ), Rio de Janeiro, RJ, Brazil; 5 Rede Micologia RJ – Fundação de Amparo à Pesquisa do Estado do Rio de Janeiro (FAPERJ), Rio de Janeiro, RJ, Brazil

**Keywords:** *Candida haemulonii*, biofilm, secreted aspartic peptidases, aspartic peptidase inhibitor, pepstatin A, HIV peptidase inhibitors

## Abstract

*Candida haemulonii* is an emerging, opportunistic, and multidrug-resistant fungal pathogen. Recently, our group reported the ability of *C. haemulonii* to form biofilm and secrete aspartic-type peptidases (Saps). Herein, we investigated the correlation between Saps production and biofilm formation along *C. haemulonii* growth in yeast carbon base medium supplemented with albumin (a Sap-inducing condition) and in the presence of the classical Sap inhibitor pepstatin A. Under these conditions, the biofilm biomass increased on a polystyrene surface, reaching its maximum at 96 h, while maximum biofilm viability was detected at 48 h. The release of Saps during biofilm formation showed an inverse trend, with the highest enzymatic activity measured after 24 h. In the presence of pepstatin A, a significant reduction in biofilm parameters (biomass and viability), as well as in albumin consumption by biofilm-forming cells was detected. These findings underscore the importance of Saps during the biofilm development in *C. haemulonii*.

## 1. Introduction

*Candida haemulonii sensu stricto* belongs to the *Candida haemulonii* clade, which also includes *C. duobushaemulonii*, *C. haemulonii* var. *vulnera*, *C. auris*, *C. pseudohaemulonii*, *C. vulturna*, and *C. khanbhai* [1]. Infections caused by this fungal species are rare but warrant attention due to their resistance to most antifungal agents used in clinical settings, particularly azoles and polyenes [2],[3]. Moreover, *C. haemulonii* is capable of forming biofilm, which is a key virulence attribute associated with increased resilience and resistance to both chemical and physical environmental stressors [4]. In this context, biofilms represent microbial communities embedded within a complex extracellular polymeric matrix, which facilitates microbial interactions, adhesion to biotic or abiotic surfaces, and shields fungal cells against immune responses and antifungal agents [5].

The production and secretion of hydrolytic enzymes, such as peptidases, phospholipases, esterases, and phytases, are essential for the degradation of key host substrates that sustain *Candida*'s nutrition, growth, proliferation, development, differentiation, adhesion, and infection [6],[7]. Among these enzymes, secreted aspartic peptidases (Saps) play a pivotal role in the pathogenesis of *Candida* species, particularly *C. albicans*, where they are extensively studied and recognized as major virulence factors. Saps facilitate critical processes such as adherence, biofilm formation, colonization, host tissue invasion, and evasion of host immune responses [6],[8]. To perform these essential biological functions, *C. albicans* encodes 10 *SAP* genes, whose expression is regulated by various environmental, host-related and microbial factors. Notably, studies have shown that *C. albicans* Saps expression, particularly Sap5 and Sap9, is highly regulated and secretion occurs automatically during biofilm formation, in contrast to planktonic growth [9]. Proteomic analyses have identified Sap5 and Sap6 as the predominant peptidases associated with *C. albicans* biofilms, highlighting their strong positive regulation, particularly during biofilm formation [10]. Recently, our research group demonstrated that planktonic *C. haemulonii* cells are capable of producing Sap-like enzymes when cultivated under Sap-inducing conditions [8]. However, no information is currently available regarding the ability of biofilm-forming *C. haemulonii* cells to secrete this specific class of multifunctional hydrolytic enzymes.

Based on these premises, the present study aimed to investigate the production of Saps during *C. haemulonii* biofilm formation under chemically defined conditions that induce the extracellular release of Saps by yeast cells. Furthermore, pepstatin A, the classical Sap inhibitor, was used to assess the enzymatic activity and its impact on biofilm formation by this emerging opportunistic fungal pathogen.

## 2. Materials and methods

### 2.1. Fungus and growth conditions

*Candida haemulonii* strain LIP*Ch*4, recovered from the finger nail of a patient at a Brazilian hospital [11], was used in all experiments. Notably, this fungal strain was selected due to its resistance to the polyene amphotericin B and the azole antifungals fluconazole, itraconazole, ketoconazole, voriconazole and posaconazole [11]. Fungal cells were cultured in yeast carbon base (YCB) medium supplemented with 0.1% bovine serum albumin (BSA; Sigma-Aldrich, USA) at 37 °C for 48 h under constant agitation (200 rpm), which are well-known conditions that induce the expression and secretion of Saps in different *Candida* species [12] including *C. haemulonii* [8]. The yeast cells were quantified using a Neubauer chamber.

### 2.2. Biofilm formation assay

Fungal cell suspensions in YCB-BSA medium (200 µL containing 10^6^ yeasts) were transferred into each well of a flat-bottom 96-well polystyrene microtiter plate, and incubated without agitation at 37 °C for up to 96 h. After 24, 48, 72 and 96 h, the culture supernatants were collected and then filtered through a 0.22-µm Millipore membrane to detect Sap activity and the albumin degradation profile [13]. Additionally, the pH value of culture supernatants was measured at each time point. In parallel, the polystyrene-adhered fungal cells were washed three times with phosphate-buffered saline (PBS, pH 7.2) to remove nonadherent cells (non-biofilm-forming fungi) and then processed to evaluate the biofilm parameters (biomass and viability) as described below. Plate wells containing only culture medium were used to set up the reader as blanks. Additionally, biofilm formation was conducted for 48 h in YCB-BSA medium, either supplemented or not with different concentrations (0, 1, 10, 50, and 100 µM) of pepstatin A, a typical aspartic peptidase inhibitor.

### 2.3. Measurement of biofilm parameters

Biofilm biomass was assessed following the protocol described by Peeters et al. [14]. Initially, adhered fungal cells were fixed with 200 µL of 99% methanol for 15 min. Subsequently, the supernatants were removed and the microtiter plate was air-dried for 5 min followed by the addition of 200 µL of a 0.4% crystal violet solution (prepared by diluting the stock solution in PBS; Sigma-Aldrich, USA) to each well. The plate was incubated at room temperature for 20 min, and afterwards the wells were washed once with PBS to eliminate excess stain and the biomass in each well was decolorized with 200 µL of 33% acetic acid for 5 min. One hundred microliters of the acetic acid solution were transferred to a new 96-well plate, and the absorbance was measured at 590 nm using a microplate reader (SpectraMax M3; Molecular Devices, USA) [15]. The metabolic activity of the biofilm was determined using a colorimetric assay capable of measuring the metabolic reduction of 2,3-bis (2-methoxy-4-nitro-5-sulfophenyl)-5-[(phenylamino) carbonyl]-2H-tetrazolium hydroxide (XTT; Sigma-Aldrich, USA) to a water-soluble brown formazan product [15]. The XTT/menadione solution was prepared by dissolving 2 mg XTT in 10 mL of pre-warmed PBS supplemented with 100 µL of a stock solution of menadione (0.4 mM in acetone). The XTT/menadione solution (200 µL) was added to the plate wells and the plate was incubated at 37 °C for 3 h in the dark. Then, 100 µL of the supernatant from each well were transferred to a new microplate and the colorimetric changes were quantified using a microplate reader at 492 nm (SpectraMax M3; Molecular Devices, USA).

### 2.4. BSA consumption during biofilm formation

The supernatants obtained from biofilms formed in the absence and presence of pepstatin A were filtered through a 0.22-µm membrane (Millipore, São Paulo, Brazil). The protein concentration was determined by the method described by Lowry et al. [16], using BSA as standard. Subsequently, the supernatants were mixed with an equal volume of sodium dodecyl sulfate-polyacrylamide gel electrophoresis (SDS-PAGE) sample buffer (125 mM Tris, pH 6.8, 4% SDS, 20% glycerol, and 0.002% bromophenol blue) containing 10% β-mercaptoethanol, followed by heating at 100 °C for 5 min. Proteins (10 µg of each sample) were analyzed on 10% SDS-PAGE by the method described by Laemmli [17]. Electrophoresis was carried out at 120 V and 120 mA for 90 min at room temperature, and the gels were silver-stained.

### 2.5. Supernatant concentration

The cell-free supernatants collected from biofilm formation were concentrated 10-fold using the Centricon ultrafiltration system (Millipore, Billerica, USA) with 10-kDa molecular weight cut-off membranes. The concentrated supernatants were used to evaluate proteolytic activity against substrates under various conditions, as outlined below.

### 2.6. Measurement of Sap activity

Sap activity was measured using the fluorogenic peptide substrate for cathepsin D [7-methoxycoumarin-4-acetyl-Gly-Lys-Pro-Ile-Leu-Phe-Phe-Arg-Leu-Lys(DNP)-D-Arg-amide] (Sigma-Aldrich) [18]. Cleavage of the fluorogenic substrate was continuously monitored in a spectrofluorometer (SpectraMax Gemini XPS, Molecular Devices, USA) using an excitation wavelength of 328 nm and an emission wavelength of 393 nm. The reaction was started by adding the substrate (2 µM) to the concentrated biofilm supernatants (50 µg protein) in a total volume of 100 µL of 50 mM sodium acetate buffer (pH 4.0), both in the absence and in the presence of pepstatin A at 10 µM. The reaction mixture was incubated at 37 °C up to 60 min. The assays were controlled for self-liberation of the fluorophore over the same time interval [8]. Additionally, the biofilm-derived supernatant with the highest Sap activity was selected to titrate the effect of pepstatin A at concentrations of 0.1, 1, and 10 µM. In parallel, the peptidase activity detected in the biofilm-derived supernatants was tested against various peptidase inhibitors from distinct peptidase classes, including lopinavir, darunavir, amprenavir, nelfinavir, and ritonavir (aspartic peptidase inhibitors), 1,10-phenanthroline (metallopeptidase inhibitor), *trans*-epoxysuccinyl-L-leucylamido-(4-guanidino)-butane (E-64; cysteine peptidase inhibitor) and phenylmethylsulfonyl fluoride (PMSF; serine peptidase inhibitor), all at a final concentration of 100 µM.

### 2.7. Measurement of serine peptidase activity

Serine peptidase activity was also measured using the substrate *N*-benzoyl-Phe-Val-Arg-pNa (Sigma-Aldrich), as this class of peptidases has previously been identified in the secreted molecules from planktonic *C. haemulonii* cells by our research group [28]. Cleavage of the substrate was continuously monitored in a spectrofluorometer (SpectraMax Gemini XPS, Molecular Devices, USA) using an excitation wavelength of 410 nm. The reaction was started by adding the substrate (100 µM) to the concentrated biofilm supernatants (50 µg protein) in a total volume of 100 µL of either phosphate-buffered saline (PBS, pH 7.2) or 50 mM sodium acetate buffer (pH 4.0), both in the absence and in the presence of 100 µM PMSF. The reaction mixture was incubated at 37 °C up to 60 min [28].

### 2.8. Statistics

All experiments were performed in triplicate, in three independent experimental sets. The results were analyzed statistically by the one-way analysis of variance (ANOVA) and Pearson correlation. All analyses were performed using the program GraphPad Prism8. In all analyses, *p* values of 0.05 or less were considered statistically significant.

## 3. Results

**Figure 1.**
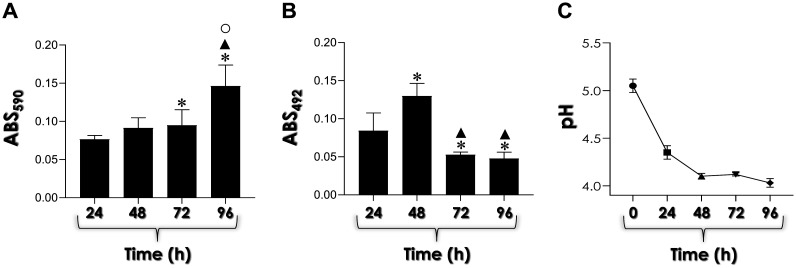
Biofilm formation by *C. haemulonii* cells under Sap-inducing conditions (YCB-BSA medium) was assessed by placing 200 µL of fungal cells (containing 10^6^ yeasts) to interact with polystyrene at 37 °C for 96 h. At each 24-h interval, the biofilm was processed to measure fungal biomass (A) by crystal violet incorporation into methanol-fixed biofilms, with absorbance measured at 590 nm. The symbols indicate statistical significance according to Tukey's multiple comparisons test (*p* < 0.0001) as follows: (*) indicates a significant difference between 24 h and either 72 h or 96 h, (▲) indicates a significant difference between 48 h and 96 h, and (○) indicates a significant difference between 72 h and 96 h. Metabolic activity (B) was assessed by measuring XTT reduction to formazan by viable cells in non-fixed biofilms at 492 nm. The symbols indicate statistical significance according to Tukey's multiple comparisons test (*p* < 0.0001) as follows: (*) denotes a significant difference between 24 h and all other time points studied, while (▲) denotes a significant difference between 48 h and either 72 h or 96 h. The pH value of the biofilm-derived supernatants (C) was monitored over time, with statistical analysis using Tukey's test indicating a significant difference at 24 h compared to all subsequent time points. The zero time point represents the pH value (5.0) of the medium before inoculation. Results are presented as mean ± standard deviation.

Initially, we assessed the biofilm formation by *C. haemulonii* cells on a polystyrene surface by measuring two classical parameters, biomass and metabolic activity, during 96 h of incubation at 37 °C in YCB-BSA medium. The results demonstrated that the kinetics of biofilm formation in *C. haemulonii* yeasts evolved over time, with the highest biomass observed at 96 h (Figure 1A) and the maximum metabolic activity recorded at 48 h (Figure 1B).

**Figure 2.**
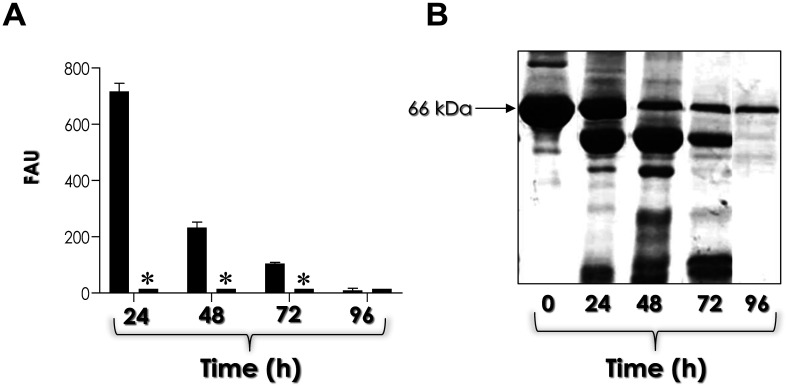
Aspartic-type peptidase activity and BSA consumption during the biofilm formation by *C. haemulonii* cells cultured under Sap-inducing conditions were evaluated. Fungal cells were grown in yeast carbon base medium supplemented with 0.1% bovine serum albumin (YCB-BSA) at 37 °C for 96 h. At each 24-h interval, the culture supernatants were collected, filtered, and used to assess aspartic peptidase activity and BSA consumption. (A) Aspartic peptidase activity was measured in each interval using the cathepsin D-specific peptide substrate in sodium acetate buffer, pH 4.0, in the absence (left bars) or in the presence (right bars) of pepstatin A at 10 µM. Proteolytic activity was quantified using spectrofluorometry with an emission at 393 nm and excitation at 328 nm. The results were expressed in fluorescent arbitrary units (FAU). Asterisks indicate significant differences at all time points between activity measured in the absence and presence of pepstatin A at 10 µM (*p* < 0.001, Tukey's multiple comparisons test). The results are presented as mean ± standard deviation. (B) BSA consumption was analyzed using SDS-PAGE, followed by silver staining. The column labeled “0” serves as the control, representing intact BSA at 66 kDa.

The pH value of the biofilm was monitored over a 96-h period. Prior to inoculation, the YCB-BSA medium had a pH of 5.0. After 24 h of fungal growth, the pH decreased to 4.4. At 48 and 72 h, the pH further dropped to 4.1, and by 96 h, it was recorded at 4.0. These results demonstrate a continuous decline in pH over time, indicating increasing acidification in the biofilm environment (Figure 1C). This acidic condition is favorable for Sap activity, as these enzymes function optimally in an acidic environment [6],[8]. In parallel, biofilm-conditioned, cell-free supernatants of *C. haemulonii* cultures were used to measure the enzymatic activity of aspartic-type peptidases at pH 4.0. Cleavage of a cathepsin D-specific fluorogenic peptide substrate demonstrated a clear decrease in pepstatin A-sensitive aspartic-type peptidase activity during biofilm maturation (Figure 2A). This result indicates a higher level of Sap activity in the early stages of biofilm formation, which is attributed to the use of BSA to facilitate this process. Consistent with this finding, significant BSA consumption was observed during the entire biofilm formation period (Figure 2B). Correlation analyses were conducted using the Pearson correlation coefficient (*r*) to assess the relationships between the analyzed biofilm parameters and Sap activity. In this regard, it was possible to observe a significant and negative correlation between biofilm biomass and Sap activity (*r* = –0.9584; *p* = 0.0416).

**Figure 3.**
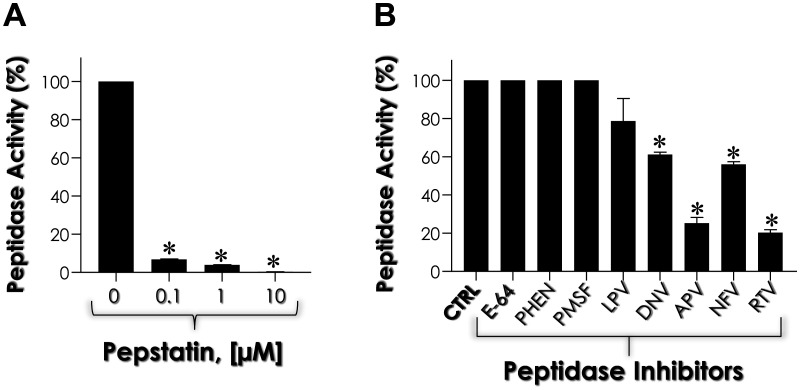
Effect of different peptidase inhibitors on the proteolytic activity of the 24-h biofilm-derived supernatant of *C. haemulonii* grown in YCB-BSA medium. (A) Impact of pepstatin A at varying concentrations (0.1 µM, 1 µM and 10 µM) on Sap activity. The results are presented as the mean percentage relative to the control, which is considered 100%. Asterisks indicate a significant difference (*p* < 0.001) between non-treated (0, control) and pepstatin A-treated systems. (B) Effect of the metallopeptidase inhibitor 1,10-phenanthroline (PHEN), the serine peptidase inhibitor phenylmethylsulfonyl fluoride (PMSF), the cysteine peptidase inhibitor *trans*-epoxysuccinyl-L-leucylamido-(4-guanidino)-butane (E-64), and HIV aspartic peptidase inhibitors (lopinavir, LPV; darunavir, DNV; amprenavir, APV; nelfinavir, NFV; ritonavir, RTV) on Sap activity. Asterisks indicate a significant difference between the control (CTRL) and aspartic peptidase inhibitor-treated systems. In both cases, results are expressed as the mean percentage relative to the control, which is set at 100%. Data are presented as mean ± standard deviation.

Our results showed that the highest enzymatic activity of aspartic-type peptidases occurred after 24 h of biofilm formation by *C. haemulonii* cells. Based on these findings, we selected this incubation period to assess the percentage of enzymatic activity inhibition using various inhibitors targeting different peptidase classes. This approach was designed to confirm that the observed protease activity specifically corresponds to the aspartic-type peptidase class. Initially, we titrated the classical aspartic peptidase inhibitor pepstatin A. The results showed that a concentration of 0.1 µM reduced Sap activity by 93.20%, 1 µM by 96.20%, and 10 µM by 99.65% (Figure 3A). Subsequently, we verified that the metallopeptidase inhibitor 1,10-phenanthroline, the cysteine peptidase inhibitor E-64, and the serine peptidase inhibitor PMSF were unable to inhibit the proteolytic activity present in the *C. haemulonii* biofilm supernatant (Figure 3B). Finally, we evaluated several human immunodeficiency virus (HIV) aspartic peptidase inhibitors at 100 µM against the biofilm-derived Saps from *C. haemulonii*. The results showed that lopinavir reduced enzymatic activity by 21.22%, darunavir by 38.79%, nelfinavir by 43.96%, ritonavir by 79.66%, and amprenavir by 74.69% (Figure 3B). Collectively, these results indicated that biofilm-forming *C. haemulonii* cells secrete aspartic peptidases. In parallel, serine peptidase activity was investigated, as this class of peptidases had previously been identified in *C. haemulonii* planktonic cells grown in Sabouraud medium. The results revealed that biofilm-derived supernatants obtained after growth in YCB-BSA medium did not exhibit serine peptidase activity capable of cleaving the peptide substrate *N*-benzoyl-Phe-Val-Arg-pNa, regardless the conditions were acidic or alkaline (data not shown).

**Figure 4.**
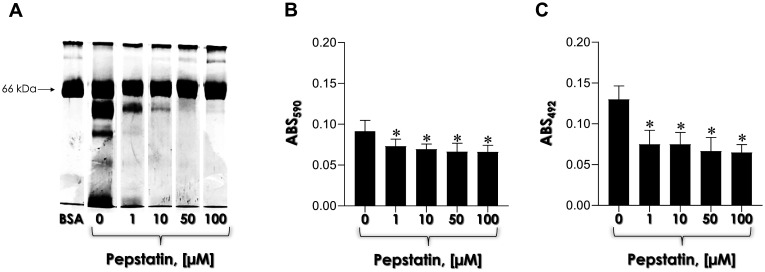
Effect of the aspartic peptidase activity on biofilm formation and BSA consumption in *C. haemulonii* cells cultured under Sap-inducing conditions. Fungal cells (200 µL containing 10^6^ yeasts) were incubated on a polystyrene surface for 48 h at 37 °C in the absence (0) and presence of varying concentrations (1, 10, 50 and 100 µM) of pepstatin A, a prototype aspartic peptidase inhibitor. Following incubation, the systems were processed to evaluate BSA (the 66-kDa band) consumption by SDS-PAGE assay (A), fungal biomass by crystal violet incorporation in methanol-fixed biofilms at 590 nm (B), and metabolic activity by the reduction of XTT to formazan by viable cells measured in non-fixed biofilms at 492 nm (C). The results are expressed as mean ± standard deviation. Asterisks denote statistically significant differences between non-treated and pepstatin A-treated systems (ANOVA, Dunnett's multiple comparison test).

Finally, pepstatin A was used to assess the potential involvement of aspartic-type peptidases in the formation of *C. haemulonii* biofilms. The results demonstrated that even at the lowest tested concentration (1 µM), pepstatin A significantly inhibited BSA consumption (Figure 4A), while also partially affecting both biofilm biomass and viability parameters, with no clear concentration-dependent effect (Figure 4B and 4C).

## 4. Discussion and conclusions

Biofilm is a complex, multimodal structure that enhances both the resistance and virulence of microorganisms [19]. In the present study, we demonstrated that the emerging fungal pathogen *C. haemulonii* is capable of forming biofilms when cultured in YCB medium supplemented with albumin as the sole nitrogenous source. Interestingly, our findings partially diverge from previous research that investigated biofilm formation kinetics using the classical Sabouraud medium [4]. In that study, Ramos et al. [4] suggested that 48 h was the optimal time interval to achieve maximum biofilm biomass formation among fungal species belonging to the *C. haemulonii* complex. In contrast, our current results indicate that 96 h is the optimal time interval for achieving the highest biofilm biomass in *C. haemulonii* cells when cultured in YCB-BSA medium. This discrepancy highlights the significant influence of nutrient availability on biofilm development. While Sabouraud medium provides a nutrient-rich environment that promotes rapid biofilm formation, the nutrient-poor YCB-BSA medium exhibited a contrasting biofilm kinetics pattern. Despite these differing nutritional contexts, both studies consistently showed that metabolic activity peaked earlier than biomass accumulation. These findings underscore *C. haemulonii*'s adaptability to diverse nutritional environments and suggest that nutrient availability plays a pivotal role in modulating biofilm dynamics.

Other studies have demonstrated that non-*albicans Candida* species tend to exhibit significantly increased biofilm formation between 48 and 96 h of incubation under various culture conditions [20],[21]. For example, Tan et al. [21] investigated the impact of different culture media, RPMI 1640, YPD and BHI, on the biofilm formation by *C. tropicalis*, *C. krusei* and *C. parapsilosis*. YPD and BHI media, known for their nutrient-rich composition, were compared to RPMI 1640, a synthetic defined medium commonly used for biofilm formation although containing relatively less nutrients. The study revealed that *Candida* species cultivated in YPD medium exhibited the highest levels of biofilm formation, while those grown in RPMI 1640 consistently showed the lowest biofilm formation. These findings highlight the critical role of nutrient composition in biofilm development in non-*albicans Candida* species, suggesting that richer media promote higher biofilm biomass production [21].

Concurrently, we investigated the enzymatic activity of aspartic-type peptidases in cell-free supernatants collected at different time points from biofilm-forming *C. haemulonii* cells. Our findings revealed a peak in pepstatin A-sensitive aspartic peptidase activity during the initial stages of biofilm formation, emphasizing the enzyme's pivotal role in this early phase. The subsequent decline in aspartic peptidase activity suggests a shift in cellular regulation as the biofilm matures, with reduced reliance on enzymatic processes critical for early establishment. The fluctuating activity of aspartic peptidases observed during biofilm maturation aligns with previous studies on *Candida* spp., in which these enzymes take part in various aspects of biofilm formation, including adhesion, nutrient acquisition, and matrix development [22]. In this context, Kadry et al. [23] established a compelling association between biofilm formation and the expression of *SAP9* and *SAP10* genes in *C. albicans* isolates. Their findings demonstrated that the majority (66.7%) of strong biofilm-producing isolates exhibited higher expression levels of both *SAP9* and *SAP10* genes, while 25% of isolates showed elevated expression of either *SAP9* or *SAP10* alone. These results suggest a potential role for Sap activity in facilitating the initial stages of biofilm formation, further reinforcing the critical involvement of aspartic peptidases in *Candida* biofilm development. Similarly, Winter et al. [10] investigated the influence of *SAP5* and *SAP6* genes, which are specifically produced during *C. albicans* biofilm formation, through both *in vitro* and *in vivo* mouse model experiments. Their study revealed that the expression levels of *SAP5* and *SAP6* in mature *in vivo* biofilms were approximately 50 times higher compared to the planktonic state. This disparity was particularly pronounced when comparing wild-type strains with their mutant counterparts. The deletion of *SAP5* and *SAP6* genes resulted in a significant reduction in *C. albicans* biofilm development in both *in vitro* and *in vivo* models, underscoring the pivotal role of Saps in biofilm formation. Relevantly, the supernatants recovered from biofilm-forming *C. haemulonii* cells grown in YCB-BSA medium displayed Saps, which were effectively inhibited by pepstatin A and partially blocked by HIV aspartic peptidase inhibitors, particularly ritonavir, amprenavir, nelfinavir, and darunavir, but not serine peptidases.

In a recent study, seven *SAP* genes were identified in *C. auris*, with *SAPA3* emerging as the primary Sap in this multidrug-resistant fungal pathogen [24]. Deletion of the *SAPA3* gene resulted in a significant reduction in Sap activity and a marked decrease in *C. auris* virulence. Notably, the *SAPA3* mutant strain exhibited a 28% decrease in biofilm formation compared to the wild-type strain. These findings highlight the pivotal role of *SAP*-like genes in biofilm formation in *C. auris*, a species that is phylogenetically closer to *C. haemulonii* [24]. Our findings are consistent with previous studies, underscoring the importance of Sap activity in the establishment of *C. haemulonii* biofilms. Furthermore, the negative correlation between biofilm biomass and aspartic peptidase activity suggests the involvement of a regulatory mechanism in biofilm formation dynamics. While *SAP*-like genes and Sap-like activity have been identified in *C. haemulonii* [8],[25], our understanding on their expression under optimal conditions remains limited. This gap in knowledge represents a key area for future research within our group, aiming to unravel the factors that influence their expression and activity, thereby providing a more comprehensive understanding of biofilm formation dynamics in *C. haemulonii*.

A significant consumption of BSA during biofilm formation by *C. haemulonii* cells was observed, underscoring the importance of nutrient acquisition mechanisms in biofilm development. BSA not only serves as a nutrient source but also acts as a potential inducer for aspartic peptidase secretion [12]. The hydrolysis of BSA generates readily available peptides and free amino acids, which are utilized by the yeast for nutritional and metabolic purposes [8],[12]. Consistent with the enzymatic activity observed in our study, a previous investigation by our research group documented a similar pattern of BSA degradation across different strains of the *C. haemulonii* species complex over a 72–96 h incubation period in YCB-BSA medium, employing the same analytical methodology [8].

In the subsequent phase, we employed pepstatin A, a well-known inhibitor of aspartic peptidases, to investigate the potential involvement of these enzymes in *C. haemulonii* biofilm formation. Notably, our results revealed a significant reduction in both biofilm biomass and viability, even at the lowest tested concentration. This effect can likely be attributed to the direct inhibition of aspartic peptidases secreted by *C. haemulonii* cells within the biofilm matrix. Since these enzymes are integral to several processes essential for biofilm formation, their inhibition likely disrupts these processes, leading to the observed decrease in both biofilm biomass and viability.

In a previous study conducted by our research group, we demonstrated the effective inhibition of BSA hydrolysis by pepstatin A when incubated with the cell-free culture supernatants of several isolates belonging to the *C. haemulonii* species complex [8]. This finding is consistent with the literature, which suggests that various compounds with peptidase inhibitor properties can disrupt *Candida* spp. biological processes by targeting their aspartic peptidases. For example, recent studies have reported similar reductions in biofilm biomass and viability in *C. albicans* strains when grown in the presence of two HIV aspartic peptidase inhibitors, atazanavir and darunavir [26]. These anti-biofilm effects were linked to the downregulation of the gene encoding Sap2, the most critical extracellular peptidase in the Sap superfamily for *C. albicans*. Furthermore, contrary to the findings reported by Consolaro et al. [27], who observed no significant difference in biofilm formation in *C. albicans* in the presence of 1 µM pepstatin A, our study demonstrated that the same concentration induced a partial reduction in *C. haemulonii* biofilm formation, with a particularly pronounced effect on cell viability. While the specific mechanisms underlying cell viability in biofilms treated with pepstatin A remain incompletely understood, it is well-established that pepstatin A is a classical inhibitor of Sap activity. This inhibitory effect likely disrupts multiple processes within biofilms, including nutrient acquisition and extracellular matrix formation. As such, the observed reduction in biofilm biomass may be attributed to a decrease in the number of viable cells, given the critical role of aspartic peptidases in both planktonic cells and biofilm architecture.

Based on the recognized importance of Saps in the pathogenicity of *Candida* spp., our findings provide valuable insights into their role during biofilm development in *C. haemulonii*. By demonstrating the impact of an aspartic peptidase inhibitor on biofilm formation, our study reinforces the essential role of Saps in this process, highlighting their potential as targets for combating *Candida* biofilm-related infections. These results contribute to a deeper understanding of fungal pathogenesis and open promising avenues for the development of innovative antifungal strategies aimed at disrupting biofilm formation and enhancing treatment efficacy.

In conclusion, this study highlights the pivotal role of Saps in *C. haemulonii* biofilm formation. Through comprehensive analyses, we observed significant correlations between biofilm biomass, viability, and Saps activity, shedding light on key mechanisms that drive *Candida* biofilm development. Notably, a statistically significant reduction in biofilm formation was observed in the presence of the aspartic peptidase inhibitor pepstatin A. Our findings not only support previous research on the pathogenicity of *Candida* spp. but also provide novel insights into potential therapeutic targets for combating biofilm-related infections, offering promising avenues for future antifungal strategies.
